# Do Temporal Eating Patterns Differ in Healthy versus Unhealthy Overweight/Obese Individuals?

**DOI:** 10.3390/nu13114121

**Published:** 2021-11-17

**Authors:** Fatin Hanani Mazri, Zahara Abdul Manaf, Suzana Shahar, Arimi Fitri Mat Ludin, Norwahidah Abdul Karim, Nur Diyana Dalila Hazwari, Qi Wen Kek, Siti Munirah Abdul Basir, Asnida Arifin

**Affiliations:** 1 Dietetic Program and Centre for Healthy Aging and Wellness, Faculty of Health Sciences, Universiti Kebangsaan Malaysia, Jalan Raja Muda Abdul Aziz, Kuala Lumpur 50300, Malaysia; fatinhananimazri@gmail.com (F.H.M.); suzana.shahar@ukm.edu.my (S.S.); diyanadalila26@gmail.com (N.D.D.H.); kekqiwen@gmail.com (Q.W.K.); sitimunirah.abdulbasir@gmail.com (S.M.A.B.); 2 Biomedical Science Program and Centre for Healthy Aging and Wellness, Faculty of Health Sciences, Universiti Kebangsaan Malaysia, Jalan Raja Muda Abdul Aziz, Kuala Lumpur 50300, Malaysia; arimifitri@ukm.edu.my; 3 Department of Biochemistry, Faculty of Medicine, Universiti Kebangsaan Malaysia Medical Centre, Cheras, Kuala Lumpur 56000, Malaysia; wahida2609@gmail.com; 4 Centre for Healthy Aging and Wellness, Faculty of Health Sciences, Universiti Kebangsaan Malaysia, Jalan Raja Muda Abdul Aziz, Kuala Lumpur 50300, Malaysia; asnidaarifin@ukm.edu.my

**Keywords:** obesity, metabolically healthy obesity, chrono-nutrition, meal timing, eating window, chronotypes, non-shift workers

## Abstract

This study examined whether the temporal patterns of energy and macronutrient intake in early and late eating windows were associated with metabolically healthy obesity (MHO) and metabolically unhealthy obesity (MUO) among non-shift workers. A total of 299 overweight/obese non-shift workers (Age: 40.3 ± 6.9 years; 73.6% women; BMI: 31.7 ± 5.0 kg/m^2^) were recruited in the Klang Valley area of Malaysia. The biochemical parameters were determined from fasting blood samples, whereas information on dietary intake and timing was obtained from a 7-day diet history questionnaire. The midpoint of eating was used to determine the early and late windows. Compared to MHO non-shift workers (*n* = 173), MUO non-shift workers (*n* = 126) had lower energy intake from carbohydrates and protein during the early window. In contrast, MUO participants had greater energy intake from carbohydrates and fat during the late window. Participants with unhealthy metabolic status (regardless of their chronotypes) had similar temporal patterns of energy intake characterized by smaller energy intake during the early window and greater energy intake during the late window compared with participants with healthier metabolic status. Overall, the lowest percentile of energy intake during the early window was associated with an increased risk of MUO, after adjustment for potential confounders [odds ratio (OR) = 4.30, 95% confidence interval (CI) 1.41–13.11]. The greater the energy intake during the late window, the greater the risk of MUO (OR = 2.38, 95% CI 1.11–5.13) (OR = 2.33, 95% CI 1.03–5.32) (OR = 4.45, 95% CI 1.71–11.56). In summary, consuming less energy earlier in the day and more energy and carbohydrate later in the day was associated with a greater risk of MUO. Thus, a prospective study is needed to explore the potential role of chrono-nutrition practices in modifying risk factors to delay the transition of MHO to MUO.

## 1. Introduction

Obesity is one of the most significant global health threats; its prevalence has rapidly tripled from 1975 to 2014 [1]. Since 1990, this global overweight/obesity increase has also affected low and middle-income countries (LMICs) [2]. In some LMICs, as well as in rural areas of East Asia, the Pacific and South Asia, the burden has doubled as these countries are faced with an increase in the overweight population without a reduction in the prevalence of being underweight [3]. Obesity is linked with increased risks of developing diabetes mellitus, hypertension, fatty liver disease, hormonal disturbance, cardiovascular disease, and cancer, and with increased mortality [4]. Nonetheless, metabolically healthy obesity (MHO) has emerged as a sub-phenotype of obesity wherein affected individuals have a lower risk of metabolic diseases and mortality than those suffering from metabolically unhealthy obesity (MUO) [5]. 

Since obesity is a multifactorial condition, in which social, environmental and genetic factors interact in the development of the metabolic disorder, a range of approaches to weight intervention have been tried [6,7,8,9]. It remains, however, a challenge for individuals with obesity to achieve a normal weight or to maintain weight loss [10]. Thus, delaying the transition of MHO to MUO is suggested as a pragmatic strategy to reduce complications from obesity [11]. MHO is a temporary state and could transition to MUO as a result of ageing, weight gain, and an unhealthy lifestyle [12]. Nutritional factors, such as energy restriction and healthy eating patterns, have been identified as potential factors associated with MHO duration [13,14]. However, little is known about the link between chrono-nutrition and MHO and MUO. 

Chrono-nutrition research focuses on temporal eating patterns, including meal timing and frequency, meal composition and distribution, and meal alignment with circadian rhythms [15,16,17]. Later meal timing has been associated with increased adiposity [18] and cardiometabolic risk factors [19] among overweight/obese adults; however, the connection to MHO and MUO has not been explored. Nevertheless, a recent study reported that MHO was associated with greater adherence to dietary recommendations, consuming a complete breakfast, greater fruit intake, and higher meal frequency, compared to MUO [20]. Among working adults, MHO was associated with lifestyle characteristics such as frequent exercise, not smoking, and avoidance of heavy drinking [21]. However, there is a paucity of evidence regarding relationships between patterns of energy and macronutrient intake throughout the day and MHO and MUO. It is crucial to assess differences in temporal eating patterns between chronotype groups and their relationship with metabolic health status (MHO and MUO) as the evening chronotype is associated with delayed mealtimes and excessive calorie intake at night compared to the morning chronotype [22,23]. Therefore, this study sought to investigate whether the temporal pattern of energy and macronutrient intake in early and late eating windows was associated with MHO and MUO among non-shift workers. In addition, the study examined the relationship between metabolic health status and chronotype with respect to temporal eating patterns. We hypothesized that smaller energy intake in the early eating window (early window) and greater energy intake in the late eating window (late window), as well as later meal timing, would be associated with MUO.

## 2. Materials and Methods

### 2.1. Study Design and Sampling Method

This cross-sectional study was conducted among overweight and obese government non-shift workers in the Klang Valley area, an urban area in the centre of Malaysia. The required sample size was determined using GPower, version 3.1. The calculation of the sample size was based on an F-test with an f effect size of 0.21(from the mean and standard deviation in a previous study [24]), an alpha level of 0.05, and a test power of 80%. A total of 327 participants was required, assuming 10% attrition. The inclusion criteria were: body mass index (BMI) ≥ 25.0 kg/m^2^ according to the Asian-Pacific cut-off point for obesity [25], age between 20 and 59 years, and non-shift employment. This study included only non-shift workers to control for variation in possible circadian misalignment caused by work schedules. The exclusion criteria were: pregnancy, current lactation, current recipient of bariatric surgery, and diagnosis of chronic disease, such as liver, heart, kidney disease, or cancer. 

The data collection was conducted from August 2019 to January 2020. Information about the research was disseminated via online posters, email, and social media (Facebook and WhatsApp). Participation was voluntary and informed consent was obtained from all participants before being recruited. A total of 369 potential participants registered, however, only 339 of these attended screening assessments. The final analysis included 299 participants. Of these, 40 were excluded for the following reasons: normal BMI (*n* = 14), submitted incomplete questionnaires (*n* = 9), shift workers (*n* = 5), withdrew (*n* = 9), and extreme data outliers (*n* = 3). The study was conducted in accordance with the Declaration of Helsinki and approved by the Research and Ethical Committee of Medical Research of the Universiti Kebangsaan Malaysia (UKM PPI/111/8/JEP-2017-656).

### 2.2. Adiposity and Biochemical Parameters

For adiposity parameters, body weight and composition were determined using a bioelectrical impedance analyser, TANITA DC-360 (Tanita Corporation of America, Arlington Heights, IL, USA), to the nearest 0.1 kg. For body weight and composition, participants were asked to remove their shoes and socks and then stand in an upright position with their feet touching the electrodes. Participants’ heights were determined using a portable stadiometer (Seca 213, Hamburg, Germany). They were asked to stand in an upright position with their head oriented in the Frankfort horizontal plane. BMI was calculated as weight (kg) divided by height squared (m^2^). Waist circumference (WC) was measured midway between the lower rib margin and the iliac crest to the nearest 0.1 cm using a -non-expandable tape measure. 

Phlebotomists drew a total of 10 mL of fasting peripheral venous blood for biochemical parameters [fasting blood glucose (FBG), insulin, HbA1c, total cholesterol, high-density-lipoprotein cholesterol (HDL-C), low-density-lipoprotein cholesterol (LDL-C), non-HDL-C, triglycerides, and uric acid]. The homeostatic model assessment for insulin resistance (HOMA-IR) was calculated from the formula: fasting blood glucose x insulin level/22.5 [26]. The participants were asked to fast for 8 h (overnight) and blood collection was conducted at 08:00. The systolic and diastolic blood pressures were measured using a calibrated digital automatic blood pressure monitor (OMRON, Osaka, Japan). 

### 2.3. Determination of Metabolic Health Status

Metabolic health status was defined based on the five criteria proposed by the Joint Interim Statement (JIS): (1) FBG ≥ 5.6 mmol/L or drug treatment; (2) fasting TG ≥ 1.7 mmol/L or drug treatment; (3) fasting HDL-C < 1.29 mmol/L (woman), <1.03 mmol/l (man); (4) systolic blood pressure ≥ 130 mmHg, diastolic blood pressure ≥ 85 mmHg or drug treatment; (5) WC ≥ 80 cm (woman), ≥90 cm (man) [27]. Metabolic healthy obesity (MHO) was defined as having two or fewer of these components while metabolic unhealthy obesity (MUO) was defined as having three or more of the five metabolic syndrome components [24]. 

### 2.4. Dietary Intake and Timing

Dietary intake was retrospectively assessed using the validated 7-day Dietary History Questionnaire (DHQ) [28] and administered by trained dietitians/nutritionists. The household measures were used to assist participants to visualise portion size estimation [29]. Participants were asked to recall their regular dietary intake. Nutrient intake was then analysed using the Nutritionist Pro™ software (Axxya Systems, Woodinville, WA, USA). The Malaysian Food Composition database was selected when analysing the nutrient intake using the Nutritionist Pro™. For food items that were not available in the Malaysian Food Composition database, other Asian food databases were used, such as the Singapore Food Composition Guide.

The participants were also asked the average timing of each meal. The midpoint of eating time was calculated from the first and last meals [19]. Based on the midpoint of eating, the temporal patterns of energy and macronutrient intake were classified into early and late eating windows. The early eating window (early window) refers to the intake before the midpoint of eating. While the late eating window (late window) refers to the intake after the midpoint of eating. From this data, the following variables were calculated:

- Energy intake (kcal) during early window = the sum of energy intake before midpoint of eating. Thus, %E intake during early window = [(energy intake (kcal) during early window ÷ total energy intake) × 100]. The same calculation method was applied for intake in the late window.
- For example, carbohydrate intake early window = the sum of carbohydrate intake before the midpoint of eating. Thus, %E from carbohydrate intake during early window = [((carbohydrate intake (g) during early window × 4 kcal) ÷ total energy intake) × 100]. The same calculation method was applied for the intake in the late window and applied to other macronutrient (e.g., protein and fat) intake.

### 2.5. Night Eating Syndrome

The Night Eating Questionnaire (NEQ) was used to assess the presence of night eating syndrome [30]. There are 17 items in the NEQ, but the total was calculated from 13 items for this study; items 1 to 12 and 14. Items 1, 4 and 14 were reverse-scored. The total NEQ score ranged from 0 to 52. Higher scores indicate a higher degree of night eating syndrome. A score of ≥25 suggests the presence of night eating syndrome. Cronbach’s alpha coefficient for the 13 items was 0.61, indicating moderate internal consistency [31]. 

### 2.6. Chronotypes and Physical Activity

As reported in our previous work, about 20% of the population exhibits a split-sleep pattern due to religious practice involving dawn prayer [32]. Thus, the chronotypes were determined using the Munich Chronotype Questionnaire (MCTQ) [33] modified for split-sleep non-shift workers, which had been validated before the current study [32]. The median midpoint of sleep on free days (corrected sleep debt) of the sample population was used to stratify participants into morning and evening chronotypes [34]. 

The validated Malay version of the Global Physical Activity Questionnaire (GPAQ) [35] was administered to assess participants’ physical activity over the past seven days, whereas the metabolic equivalent of task (METs) was used to determine the level of physical activity [36].

### 2.7. Statistical Analysis

Normality was confirmed using histograms and skewness and kurtosis level. For the socio-demographic characteristics, an independent t-test and chi-square test were performed for continuous and categorical variables, respectively. Analysis of covariance (ANCOVA) was used to assess the differences in adiposity and biochemical parameters, lifestyle characteristics, dietary intakes, and temporal patterns of energy and macronutrient intake between MHO and MUO, adjusted for age, gender, and BMI. A two-way ANOVA was used to examine the interaction between metabolic health status (MHO and MUO) and chronotypes (morning and evening chronotypes) on lifestyle characteristics, dietary intake, and temporal pattern of energy and macronutrient intake, adjusted for age, gender, and BMI. Logistic regression models were tested to assess the temporal patterns of energy and macronutrient intake associated with the risk of being MUO. In model 1, the analyses were adjusted for age and gender. The analyses were further adjusted for the potential confounding variables, BMI and total energy intake, in model 2. The regression was also performed using Model 2 within each chronotype. Statistical analyses were conducted using the Statistical Package for Social Science (SPSS, Version 22.0, IBM, Armonk, NY, USA). The level of significance was set at *p* ≤ 0.05.

## 3. Results

Approximately 57.9% of participants were classified as MHO (*n* = 173) and 42.1% as MUO (*n* = 126). Both groups were of similar mean age but there was a significantly higher percentage of men who were classified as MUO (53.2%) compared to women (38.2%) (Table 1). MUO participants had poorer adiposity and biochemical parameters than MHO participants, except for total cholesterol, LDL-cholesterol, and uric acid levels. MUO also had higher systolic and diastolic blood pressure than MHO. There was no significant difference in the distribution of MHO and MUO between morning and evening chronotypes.

Table 2 shows there were no differences between MHO and MUO in all sleep traits, physical activity level and night eating syndrome score. In terms of total dietary intakes, there was no significant difference in total energy and macronutrient intake between MUO and MHO when adjusted for age, gender, and BMI. The total dietary intakes were also analysed within gender (Supplementary: Table S1). However, no significant difference was observed in energy and macronutrient intake between MHO and MUO within each gender (Supplementary: Table S1). Although the total eating window (hour) and meal timing (first and last meal) were similar between the metabolic health status types, MHO reported delay in the midpoint of eating compared to MUO (14:36 ± 0:56 vs. 14:21 ± 0:53, *p* = 0.037). However, no significant difference was observed in elapsed time between the last meal and sleep onset between MHO and MUO participants.

Table 3 shows the interaction effect between metabolic health status and chronotypes on the lifestyle characteristics and dietary intakes. There was no significant interaction effect observed for all sleep traits, physical activity level, night eating syndrome score, total dietary intake, and meal timing, adjusted for the potential confounding variables. This indicated that the lifestyle characteristics practiced by MHO and MUO were not significantly different between morning and evening chronotypes.

**Figure 1:**
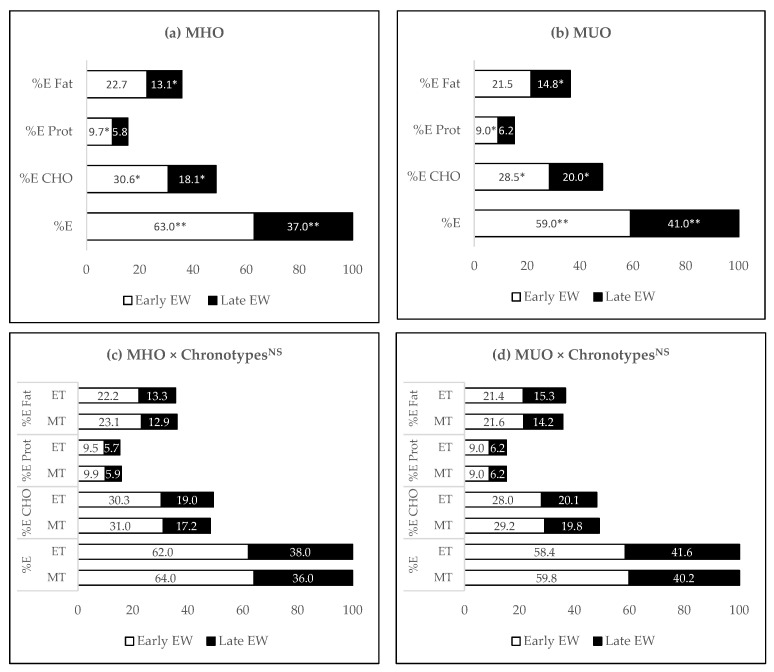
The temporal patterns of intake—the distribution of percentage total energy and percentage of energy from macronutrients (carbohydrate, protein, and fat) in the early and late windows. (**a**) MHO (**b**) MUO (**c**) MHO × chronotypes and (**d**) MUO × chronotypes. * Significant difference between MHO and MUO at *p* < 0.05, ** *p* < 0.01 using ANCOVA adjusted for age, gender, and BMI. ^NS^ No significant interaction effect of metabolic health status and chronotypes observed using two-way ANOVA adjusted for age, gender, and BMI. Abbreviations: MHO, metabolically healthy obesity; MUO, metabolically unhealthy obesity; E, energy; CHO, carbohydrate; Prot, protein; M, morning chronotypes; E, evening chronotypes.

The temporal patterns of energy and macronutrient intake based on eating windows (early and late windows) are shown in Figure 1. Compared with MHO, MUO participants consumed a lower proportion of energy (59.0 vs. 63.0%, *p* = 0.008), carbohydrate (28.5 vs. 30.6%, *p* = 0.021), and protein (9.0 vs. 9.7%, *p* = 0.049) during the early window, and more energy (37.0 vs. 41.0%, *p* = 0.008), carbohydrate (20.0 vs. 18.1%, *p* = 0.019), and fat (14.8 vs. 13.1%, *p* = 0.024) during the late window (Figure 1a,b).

Further analysis of the interaction between metabolic health status and chronotype on temporal pattern of energy and macronutrient intake in the early and late windows showed no significant association (Figure 1c,d). This indicated that the distribution of energy and macronutrient consumption according to eating windows in MHO and MUO participants was similar for morning and evening chronotypes. Both morning and evening chronotypes identified as MHO consumed greater than 60% of energy during the early window. In contrast, both morning and evening chronotypes that were identified as MUO had less than 60% of energy during the early window and greater than 40% of energy intake during the late window.

Table 4 shows the logistic regression of the risk of MUO based on the percentile intake of energy and selected macronutrients in the early and late windows. The lowest percentile of energy intake (less than 893 kcal) during the early window was significantly associated with a 4 times greater risk of being MUO compared to the intake in the highest percentile, after controlling for individual differences in age, gender, BMI, and total energy intake (95% CI 1.40–12.53, *p* = 0.011). Energy intake in the late window of more than 512 kcal was significantly associated with between 2.38 to 4.45 times greater risk of being MUO compared to the intake in the lowest percentile (95% CI 1.11–5.13, *p* = 0.027; 95% CI 1.03–5.32, *p* = 0.044; 95% CI 1.71–11.56, *p* = 0.002). Carbohydrate intake in the late window greater than 109.5 g was also significantly associated with a higher risk of MUO compared to the intake in the lowest percentile (OR = 3.28, 95% CI 1.31–8.19, *p* = 0.011). A fat intake in the late window greater than 36.4 g was associated with a 2.28 times higher risk of MUO (95% CI 1.14–4.56, *p* = 0.020), but the association was not significant after further adjustment for BMI and total energy intake.

Further analysis of the risk of MUO was stratified based on chronotypes (Table 4). The lowest percentile of energy intake during the early window was significantly associated with an 8 times greater risk of being MUO compared to the intake in the highest percentile only among morning chronotypes (95% CI 1.57–44.86, *p* = 0.013). Moreover, an energy intake during the late window greater than the 75th percentile was associated with 3.69 to 4.85 times greater risk of being MUO among morning chronotypes (95% CI 1.18–11.58, *p* = 0.025; 95% CI 1.44–16.39, *p* = 0.011). The highest percentile of carbohydrate intake in the late window was also associated with 5.56 greater odds of being MUO compared to the intake in the lowest percentile only among morning chronotypes (95% CI 1.47–21.02, *p* = 0.011). In contrast, the odds of being MUO did not significantly differ between the highest and lowest percentile of energy intake in the late window among evening chronotypes [OR 4.53 95% CI 0.87–23.58, *p* = 0.073]. There was also no significant association between energy intake in the early window and risk of MUO among evening chronotypes.

## 4. Discussion

In this study, the temporal patterns of energy and macronutrient intake were the differentiating factors between MHO and MUO. A smaller intake during the early window coupled with a greater intake during the late window was significantly associated with a greater risk of MUO among overweight/obese non-shift workers, though the total daily energy intake between MHO and MUO were similar. Furthermore, we discovered that both morning and evening chronotypes with unhealthy metabolic status, practiced similar temporal patterns of energy intake compared to the chronotypes with healthier metabolic status (Figure 1c,d). In line with this finding, both chronotypes displayed an equal proportion of MHO and MUO suggesting that both chronotypes presented a similar risk of metabolic derangement when the temporal eating patterns were against the circadian rhythm.

Our study suggests that smaller energy consumption during the earlier part of the day and greater energy consumed during the later part of the day was associated with a greater risk of unhealthy metabolic status. Consistent with the findings in this study, a one-year prospective study among adult females reported that a higher percentage of daily calories consumed during evening meals (dinner and supper) were associated with worsening diastolic blood pressure [37]. Furthermore, two randomised controlled trials involving weight loss intervention found that participants with the highest energy intake during earlier meals (breakfast and lunch) with reduced energy intake during later meals (dinner) had the highest weight loss and reduction in insulin resistance [38,39]. These studies would have been more informative if the chronotypes had been explored. This is because our study found that evening chronotypes with healthier metabolic status had greater energy intake during the early window and less during the late window than evening chronotypes with unhealthy metabolic status (Figure 1c,d). Similar temporal eating patterns were observed among morning chronotypes. Our findings suggest that greater energy consumption during the earlier part of the day and smaller consumption during the later part of the day are metabolically beneficial for morning and evening chronotypes. However, temporal eating patterns against the circadian norm are equally detrimental to both chronotypes. These results are similar to those reported by a 7-year follow-up study [40]. At baseline, evening chronotypes displayed significant greater energy intake after 20:00 compared to morning and intermediate chronotypes. During the follow up, the highest quartile of energy intake after 20:00 was associated with increased BMI, but no significant difference in risk was observed between the chronotypes. This finding indicates that greater evening energy intake was associated with obesity regardless of the chronotypes. In contrast, a cross-sectional study among obese adults reported that eveningness (towards evening chronotypes) was associated with greater energy intake after 20:00, higher BMI, and lower HDL-cholesterol [41]. Initial cross-sectional observations suggest that there may be a link between evening chronotypes and greater food consumption towards the later part of the day, possibly due to delay in meal timing. An hour delay in mealtime was associated with 53 kcal greater energy intake, higher glycaemic load, higher eating frequency and waist circumference among overweight/obese adults [42]. Despite that, the longitudinal study showed that all the chronotypes had an equal risk of weight gain from high-calorie evening meals. There would therefore seem to be a definite need for randomized control trial study to further investigate the connection between the temporal pattern of energy intake and metabolic health status in relation to the chronotypes.

In the chronotypes stratified analysis, our results suggest that morning chronotypes who consumed the least during the earlier part of the day were at risk of consuming more during the later part of the day and this temporal eating pattern was more likely to be associated with unhealthy metabolic status. However, the energy intake in the 100th percentile was associated with four times the risk among evening chronotypes, but the association was not significant (*p* = 0.073). Taken together, these results suggest that eating more towards the end of the day may be more deleterious to morning chronotypes than evening chronotypes. There are several possible explanations for this result. The possible mechanism could be related to the proximity of the last mealtime and calorie consumption to melatonin onset [43,44]. Greater energy intake closer to the biological circadian phase—dim light melatonin onset (DLMO)—was linked to higher percent body fat [43,44]. In our study, morning chronotypes had shorter elapsed time between the last meal and sleep onset compared to evening chronotypes (2.6–2.8 h vs. 3.1–3.5 h), but no significant difference was observed in relation to metabolic health status. Despite this, this finding is limited due to the lack of an objective measure of sleep traits whereas, the previous studies [43,44] used DLMO which is an established marker of circadian phase. Furthermore, with a smaller sample size in the chronotypes stratified analysis (and a wider confidence interval), the results needs to be interpreted with caution, as there might be insufficient power for a significant risk of MUO in evening chronotypes to be detected. In contrast to our findings, a larger cross-sectional study (N = 872) conducted among normal weight, overweight, and obese adults, found that the highest percentile of energy intake within 2 h prior to sleep was associated with a greater risk of obesity only among later chronotypes [34]. This discrepancy, though, could also be attributed to disparities in temporal patterns of the eating windows and the targeted sample population. Since these findings have not been observed elsewhere, a prospective study is needed to further investigate the interplay of chronotypes in the temporal pattern of energy intake related to metabolic health outcomes because a cross-sectional study does not enable causal inference.

Contrary to our hypothesis, we found that MHO participants had a significantly later midpoint of eating than MUO participants. MHO participants also displayed delay in the first and last mealtimes compared to MUO but the differences were not significant. There are several possible explanations for this discrepancy. First, this finding might be due to the equal proportion of MHO and MUO morning and evening chronotypes in our study. This is because the chronotypes stratified analysis revealed that evening chronotypes had later first and last mealtime and midpoint of eating compared to morning chronotypes, regardless of their metabolic health status (MHO and MUO), and vice versa. In evening chronotypes, the delayed mealtime may reflect the delay in their underlying circadian rhythm timing [23]. Both meal timing and chronotypes are genetically influenced, with the highest heritability of 64% seen for the midpoint of eating timing, and 43% for chronotypes [45]. A significant genetic overlap between meal timing and chronotypes has been found [45]. Nevertheless, late eating is a concern, as it was associated with greater total energy intake [46] and yet lower morning energy intake [47], which could lead to weight gain. Despite this, both MHO and MUO participants in our study had similar total daily energy intake. Although evening chronotypes showed a later midpoint of eating than morning chronotypes in the MHO group, they also had greater energy intake during the early window, comparable to morning chronotypes (62% vs. 64%, Figure 1c). Our findings also suggest that the intensity effect of a later mealtime might depend on the amount of food consumed at that mealtime. Even though MUO participants had an earlier midpoint of eating, they displayed lower energy intake in the earlier part of the day and greater energy intake towards the later part of the day than MHO. The impact of delay in meal timing on metabolic health might be influenced by the total energy intake and expenditure, the quantity of the food intake at the particular mealtime, and the meal timing in relation to solar clock and biological clock time [48]. Later mealtime was associated with worsened adiposity parameters [19,37]; however, to what extent this delay in mealtime affects metabolic health remains unclear due to methodological differences in meal timing studies. In a prospective study among women, each 30-min delay in the first mealtime was associated with 0.27 greater inches of waist circumference, except for the last meal [37]. A weight loss intervention study reported that late eaters (midpoint of eating after 14:30) lose significantly less weight compared to early eaters (midpoint of eating before 14:30), independent of total energy intake [19]. The late eaters had a 58 min later midpoint of eating than the early eaters group (15:25 vs. 14:27, *p* < 0.001). Whereas the difference in midpoint of eating between MUO and MHO in our study was only 15 min (14:21 vs. 14:36, *p* = 0.037). The lack of a clear cut-off signifying when the eating window should start and end to benefit metabolic health implies the need for more studies to explore the chrono-nutrition field. Furthermore, the genetic study implied a notable connection between meal timing and chronotypes [45]. Thus, a long-term follow-up study is another possible area of future research to investigate differences in meal timing between chronotypes concerning metabolic health.

Overall, findings from our study showed that temporal patterns of caloric consumptions against circadian timing were associated with greater risks of being MUO. Eating during the biological night, when we are supposed to sleep, disturbs the circadian clock system, affecting the metabolic and digestive process as many of these oscillate in a circadian manner [49]. Greater energy intake during the later part of the day is unfavourable because fewer calories are burned moving towards the end of the day. Diet-induced-thermogenesis (DIT) is generally lower after dinner than breakfast regardless of the calorie intake [50,51,52]. A randomised cross-over trial showed that DIT after evening meals was lower than after morning meals (20:00, 237 kcal vs. 08:00, 328 kcal, *p* = 0.003), given the same meal composition [51]. The decrease in DIT reflects a reduction in energy expenditure from digestion, absorption, and metabolism of the nutrients ingested [53]. Thus, persistent greater energy consumption during the later part of the day could induce weight gain.

Another key finding in this study was that greater energy from carbohydrates during the late window was also associated with an up to three times greater risk of being MUO. Similar to DIT, oxidation of the substrates displays a diurnal pattern with the highest carbohydrate oxidation during the morning and the lowest in the evening [54]. In contrast, lipid oxidation was higher in the evening than the morning, which could also imply impaired glucose tolerance following an evening meal [55,56]. Moreover, plasma glucose concentration also exhibits circadian rhythmicity, with lower glucose levels in the morning compared to the evening [57]. Post-prandial glucose was reported to be 17% higher in the evening (20:00) than the morning (08:00), independent of food intake [56]. This might be due to lower beta-cell function in the evening compared to the morning, which suggests reduced glucose tolerance during evening meals than morning mealtimes [56,58,59]. In relation to our study, the worse biochemical parameters, particularly glucose profile (higher fasting blood glucose, HbA1c, and insulin resistance), among MUO compared to MHO, possibly arise from their smaller energy intake in the earlier part of the day and yet greater energy intake towards the later part of the day, particularly from carbohydrates. Therefore, a healthy metabolic state could be achieved by eating more earlier in the day and eating less later to align the eating pattern with the physiological activities that oscillate in a circadian manner.

Our findings highlight the crucial aspect of chrono-nutrition which could be a potential adjunct strategy to improve metabolic health and thus delay the transition from MHO to MUO. Temporal patterns of caloric consumption focusing on greater energy during the first half of the day, and low energy, nutrient-dense, food in the second half of the day as our body approaches the circadian resting phase, are metabolically beneficial and applicable for both morning and evening chronotypes. However, a challenge of this strategy is the increase in food accessibility and availability in the modern world which enables increased calorie intake around the clock [60]. Furthermore, around the world, including in Malaysia, there are abundant 24-h eateries, particularly in urban settings, which encourage people to eat late at night or early in the morning [61,62]. The Malaysian Community Salt Survey (MyCoSS) discovered that almost 64.4% of adults in the survey reported eating out for at least one meal a day [63]. Most of the outside food (food away from home) was calorie-dense, high in sodium and fat, and with lower micronutrient value [64]. Therefore, our study also suggests a need to revise 24-h eateries policy as it not only prolongs available eating hours but also encourages consumption of more calories, carbohydrates, and fats, late at night, which ultimately compromises the circadian clock system.

This is a distinctive study highlighting the connection between metabolic health status and temporal eating patterns with chronotypes among overweight/obese adults. The present findings suggest the potential of chrono-nutrition strategies to delay the transition towards MUO or revert to MHO. Furthermore, this study identified individuals with split sleep in chronotype determination using a validated questionnaire for local use. This categorisation might improve precision by correcting the total sleep duration on free days that could be underestimated among individuals with split sleep. This study was specifically conducted among non-shift workers to control for variation in possible circadian misalignment caused by work schedules. However, some limitations should be noted in interpreting the present results. This is a cross-sectional study design and thus cannot be used to draw causal inferences. Prospective and intervention studies are needed to further examine the association. Further, most of the participants were women (73.6%) and of Malay ethnicity (99%). This study was conducted among government workers who are predominantly Malay. Even though the sample used did not precisely represent the general population, variation in culturally influenced dietary habits was reduced. Thirdly, the dietary intake and timing, as well as sleep parameters, were self-reported, which may have created reporting bias. Future studies may consider using objective measures such as a food diary app and accelerometer for sleep traits. There were also more obese participants in MUO than MHO. Thus, the statistical analysis was adjusted for BMI. Lastly, the participants reported the average meal timing instead of actual meal timing according to work and free days. Therefore, eating jetlag—the discrepancy in meal timing between work and free days— could not be determined in this study.

## 5. Conclusions

The findings demonstrate that temporal eating patterns differed between MHO and MUO individuals. Smaller energy intake during the early window and greater energy intake during the late window were associated with a greater risk of MUO. Even though MUO participants had a slightly earlier midpoint of eating than MHO participants, MUO participants had smaller energy intake during the earlier part of the day yet greater energy intake towards the later part of the day. Collectively, both timing and quantity of energy intake need to be aligned with circadian rhythms as they regulate most of our physiological activities. We found that morning and evening chronotypes with healthier metabolic status consumed more energy during the early window and less energy during the late window compared to the chronotypes with unhealthy metabolic status. This finding is key, as it suggests that eating more during the earlier part of the day and eating less later are metabolically beneficial and practical for both chronotypes. Thus, a prospective study is needed to explore the potential role of chrono-nutrition practices in modifying risk factors to delay the transition of MHO to MUO.

## Figures and Tables

**Table 1:** General characteristics of MHO and MUO.

Characteristics	Total (N = 299)	MHO (*n* = 173)	MUO (*n* = 126)	*p* Value
**Age ^a^**	40.3 ± 6.9	39.8 ± 6.8	41.2 ± 6.9	0.078
**Gender ^b^**				
Women, *n* (%)	220 (73.6)	136 (61.8)	84 (38.2)	0.021 *
Men, *n* (%)	79 (26.4)	37 (46.8)	42 (53.2)	
**Marital status**				
Married, *n* (%)	256 (85.6)	146 (57.0)	110 (43.0)	0.479
Unmarried, *n* (%)	43 (14.4)	27 (62.8)	16 (37.2)	
**Ethnicity ^b^**				
Malay	296 (99.0)	172 (58.1)	124 (41.9)	0.387
Chinese	3 (1.0)	1 (33.3)	2 (66.7)	
**Chronotypes ^b^**				
M-type, *n* (%)	149 (49.8)	88 (59.1)	61 (40.9)	0.675
E-type, *n* (%)	150 (50.2)	85 (57.9)	65 (43.3)	
**Smoking status ^b^**				
Non-smoker/Ex-smoker, *n* (%)	280 (96.6)	163 (58.2)	117 (41.8)	0.252
Smoker, *n* (%)	10 (3.4)	4 (40.0)	6 (60.0)	
**Hypertension, *n* (%) ^b^**	39 (14.6)	10 (25.6)	29 (74.4)	<0.001 ***
**Diabetes mellitus, *n* (%) ^b^**	15 (5.6)	1 (6.7)	14 (93.3)	<0.001 ***
**Dyslipidaemia, *n* (%) ^b^**	30 (11.2)	8 (26.7)	22 (73.3)	<0.001 ***
**Adiposity parameters** ^c^				
Weight (kg)	80.89 ± 15.87	77.62 ± 1.01	86.28 ± 1.19	<0.001 ***
BMI (kg/m^2^)	31.59 ± 4.92	30.62 ± 0.36	33.24 ± 0.42	<0.001 ***
Body fat (%)	40.83 ± 7.58	39.81 ± 0.39	42.13 ± 0.46	<0.001 ***
WC (cm)	96.62 ± 12.17	94.62 ± 0.81	101.19 ± 0.96	<0.001 ***
**Biochemical parameters ^c^**				
FBG ᶧ (mmol/L)	5.04 ± 1.15	4.75 ± 0.11	5.96 ± 0.13	<0.001 ***
Insulin ᶤ (μIU/mL)	11.55 ± 7.16	9.76 ± 0.56	15.52 ± 0.66	<0.001 ***
HbA1c (%) ᶧ	5.72 ± 0.91	5.52 ± 0.08	6.38 ± 0.09	<0.001 ***
HOMA-IR ᶤ	2.70 ± 2.41	2.06 ± 0.19	4.24 ± 0.22	<0.001 ***
Total cholesterol (mmol/L)	5.05 ± 0.92	5.08 ± 0.07	5.08 ± 0.08	0.999
Triglyceride ᶧ (mmol/L)	1.27 ± 0.78	1.03 ± 0.05	1.72 ± 0.06	<0.001 ***
LDL-C (mmol/L)	3.18 ± 0.80	3.19 ± 0.06	3.17 ± 0.08	0.828
Non-HDL-C (mmol/L)	3.75 ± 0.88	3.66 ± 0.07	3.92 ± 0.08	0.012 *
HDL-C (mmol/L)	1.30 ± 0.30	1.41 ± 0.02	1.15 ± 0.02	<0.001 ***
Uric acid (mmol/L)	0.36 ± 0.09	0.36 ± 0.01	0.37 ± 0.01	0.085
**Blood pressure ^c^**				
Systolic (mmHg)	123.67 ± 15.64	122.95 ± 1.12	129.93 ± 1.41	<0.001 ***
Diastolic (mmHg)	79.45 ± 12.99	76.58 ± 0.94	85.24 ± 1.1	<0.001 ***

^a^ Data are shown as mean ± standard deviation according to independent *t*-test. ^b^ Data are shown as number (%) according to Chi-square test. ^c^ Data are shown in mean ± standard error according to ANCOVA test, adjusted for age and gender. ᶧ inverse transform. ᶤ log transform. * Significant *p* < 0.05, *** *p* < 0.001. Abbreviations: MHO, metabolically healthy obesity; MUO, metabolically unhealthy obesity; BMI, body mass index; WC, waist circumference; FBG, fasting blood glucose; HOMA-IR, homeostatic model assessment of insulin resistance; LDL-C, low-density lipoprotein cholesterol; NHDL-C, non-high-density lipoprotein cholesterol; HDL-C, high-density lipoprotein cholesterol. Bold: classifying a groups of variables/parameters within the columns.

**Table 2:** Lifestyle characteristics and dietary intake of MHO and MUO.

Characteristics	Total (N = 299) Mean ± SD	MHO (*n* = 173) Mean ± SE	MUO (*n* = 126) Mean ± SE	*p* Value
**Sleep traits**				
Sleep duration workdays (hour)	6.2 ± 1.0	6.1 ± 0.1	6.2 ± 0.1	0.530
Sleep duration free days (hour)	6.7 ± 1.3	6.7 ± 0.1	6.6 ± 0.1	0.567
MSW (local time)	02:30 ± 0:44	02:28 ± 0:46	02:33 ± 0:41	0.227
MSF (local time)	02:55 ± 0:59	02:53 ± 1:01	02:57 ± 0:56	0.531
Social jetlag (minute)	30.9 ± 35.5	33.3 ± 2.7	27.6 ± 3.2	0.183
**Physical activity**				
MET	2203 ± 3190	1969 ± 250	2521 ± 293	0.162
**Night eating syndrome**				
Score	10.3 ± 4.8	10.2 ± 0.4	10.5 ± 0.5	0.635
**Total dietary intakes**				
Energy intake (kcal/day)	1850 ± 473	1819 ± 34	1893 ± 40	0.166
Energy intake (kcal/kg BW)	23.2 ± 6.2	23.0 ± 0.5	23.5 ± 0.5	0.453
CHO (g/day)	226.3 ± 66.7	222.3 ± 4.8	231.7 ± 5.6	0.212
% E from CHO	48.7 ± 6.5	48.9 ± 0.5	48.4 ± 0.6	0.457
Protein (g/day)	69.8 ± 17.2	69.2 ± 1.3	70.6 ± 1.5	0.480
% E from protein	15.3 ± 2.7	15.4 ± 0.2	15.2 ± 0.2	0.479
Fat (g/day)	74.0 ± 22.0	72.6 ± 1.6	76.0 ± 1.9	0.188
% E from fat	35.8 ± 5.4	35.6 ± 0.4	36.1 ± 0.5	0.511
**Meal timing**				
First mealtime (local time)	08:26 ± 0:51	08:31 ± 0:52	08:19 ± 0:48	0.099
Last mealtime (local time)	20:34 ± 1:30	20:41 ± 1:31	20:23 ± 1:28	0.110
Midpoint of eating (local time)	14:30 ± 0:55	14:36 ± 0:56	14:21 ± 0:53	0.037 *
Elapsed time between last meal and sleep onset (hour)	3.0 ± 1.5	2.8 ± 0.1	3.2 ± 0.1	0.070
Total eating window (hour)	12.1 ± 1.6	12.2 ± 0.1	12.1 ± 0.1	0.571

* Significant *p* < 0.05 using ANCOVA test adjusted for age, gender, and BMI. Abbreviations: MHO, metabolically healthy obesity; MUO, metabolically unhealthy obesity; SD, standard deviation; SE, standard error; BW, body weight; CHO, carbohydrate; % E, percentage energy; MSW, midpoint of sleep on workdays; MSF, midpoint of sleep on free days; MET, metabolic equivalent. Bold: classifying a groups of variables/parameters within the columns.

**Table 3:** Lifestyle characteristics and dietary intake according to metabolic health status and chronotype.

Characteristics	MHO (*n* = 173)		MUO (*n* = 126)		*p* Value
	M-Type (*n* = 88)	E-Type (*n* = 85)	M-Type (*n* = 61)	E-Type (*n* = 65)	
**Sleep traits**					
Sleep duration workdays (hour)	6.3 ± 0.1	6.0 ± 0.1	6.4 ± 0.1	6.1 ± 0.1	0.810
Sleep duration free days (hour)	6.9 ± 0.1	6.5 ± 0.1	7.1 ± 0.2	6.2 ± 0.2	0.095
MSW (local time)	02:03 ± 0:38	02:53 ± 0:39	02:07 ± 0:29	02:57 ± 0:35	0.965
MSF (local time)	02:07 ± 0:39	03:41 ± 0:40	02:12 ± 0:31	03:40 ± 0:37	0.492
Social jetlag (minute)	20.1 ± 3.4	47.2 ± 3.5	12.7 ± 4.2	41.2 ± 4.0	0.850
**Physical activity**					
MET	2035 ± 349	1901 ± 356	2367 ± 420	2664 ± 404	0.567
**Night eating syndrome**					
Score	9.9 ± 0.5	10.6 ± 0.6	10.3 ± 0.6	10.7 ± 0.6	0.801
**Total dietary intakes**					
Energy intake (kcal/day)	1760 ± 47	1882 ± 47	1864 ± 56	1919 ± 54	0.505
Energy intake (kcal/kg BW)	22.2 ± 0.6	23.8 ± 0.6	22.8 ± 0.8	24.2 ± 0.7	0.828
CHO (g/day)	213.5 ± 6.6	231.5 ± 6.8	228.5 ± 8.0	234.6 ± 7.7	0.403
% E from CHO	48.7 ± 0.7	49.2 ± 0.7	49.0 ± 0.8	47.7 ± 0.8	0.210
Protein (g/day)	67.6 ± 1.7	70.9 ± 1.8	70.6 ± 2.1	70.6 ± 2.0	0.408
% E from protein	15.7 ± 0.3	15.2 ± 0.3	15.3 ± 0.4	15.1 ± 0.3	0.655
Fat (g/day)	70.5 ± 2.2	74.8 ± 2.3	74.4 ± 2.7	77.4 ± 2.6	0.798
% E from fat	35.7 ± 0.6	35.6 ± 0.6	35.8 ± 0.7	36.3 ± 0.7	0.585
**Meal timing**					
First mealtime (local time)	08:19 ± 0:51	08:44 ± 0:51	08:14 ± 0:50	08:24 ± 0:47	0.151
Last mealtime (local time)	20:20 ± 1:22	21:03 ± 1:36	20:10 ± 1:14	20:36 ± 1:38	0.433
Midpoint of eating (local time)	14:19 ± 0:47	14:54 ± 0:59	14:11 ± 0:45	14:30 ± 1:00	0.184
Elapsed time between last meal and sleep onset (hour)	2.6 ± 0.2	3.1 ± 0.2	2.8 ± 0.2	3.5 ± 0.2	0.523
Total eating window (hour)	12.0 ± 0.2	12.4 ± 0.2	11.9 ± 0.2	12.2 ± 0.2	0.832

Data are shown in mean ± standard error using two-way ANOVA adjusted for age, gender, and BMI. *p* values indicated no significant interaction effects between metabolic health (MHO and MUO) and chronotypes (evening and morning chronotypes). Abbreviations: M-type, morning chronotypes; E-type, evening chronotypes; MHO, metabolically healthy obesity; MUO, metabolically unhealthy obesity; BW, body weight; CHO, carbohydrate; % E, percentage energy; MSW, midpoint of sleep on workdays; MSF, midpoint of sleep on free days; MET, metabolic equivalent. Bold: classifying a groups of variables/parameters within the columns.

**Table 4:** The temporal patterns of energy and macronutrient intake associated with MUO.

	Percentile (Intake)	Range	Risk of MUO [OR (95% CI)]			
			Overall (*n* = 299)		M-Type (*n* = 149)	E-Type (*n* = 150)
			Model 1	Model 2	Model 2	Model 2
EI Early EW	25th	<893 kcal	1.05 (0.52, 2.11)	4.30 (1.41, 13.11) *	8.40 (1.57, 44.86) *	2.68 (0.55, 12.98)
	50th	893–1165 kcal	0.85 (0.43, 1.70)	2.24 (0.88, 5.69)	3.24 (0.79, 13.30)	1.81 (0.51, 6.42)
	75th	1166–1384 kcal	0.75 (0.38, 1.51)	1.28 (0.58, 2.85)	1.31 (0.40, 4.29)	1.23 (0.40, 3.76)
	100th	>1384 kcal	1.00 (reference)	1.00 (reference)	1.00 (reference)	1.00 (reference)
CHO Early EW	25th	<107.0 g	0.74 (0.37, 1.46)	1.79 (0.68, 4.75)	1.95 (0.48, 7.97)	1.85 (0.46, 7.48)
	50th	107.0–139.0 g	0.90 (0.45, 1.79)	1.82 (0.77, 4.30)	1.82 (0.52, 6.42)	1.87 (0.57, 6.16)
	75th	139.1–171.3 g	0.51 (0.26, 1.02)	0.66 (0.31, 1.42)	0.42 (0.14, 1.28)	0.96 (0.32, 2.82)
	100th	>171.3 g	1.00 (reference)	1.00 (reference)	1.00 (reference)	1.00 (reference)
Protein Early EW	25th	<35.4 g	1.29 (0.64, 2.58)	2.45 (0.99, 6.04)	1.92 (0.55, 6.68)	3.33 (0.84, 13.30)
	50th	35.4–43.0 g	0.93 (0.47, 1.85)	1.62 (0.73, 3.61)	1.43 (0.49, 4.20)	1.80 (0.53, 6.16)
	75th	43.1–51.2 g	0.99 (0.50, 1.97)	1.19 (0.58, 2.55)	0.54 (0.18, 1.64)	2.33 (0.77, 7.04)
	100th	>51.2 g	1.00 (reference)	1.00 (reference)	1.00 (reference)	1.00 (reference)
EI Late EW	25th	<512 kcal	1.00 (reference)	1.00 (reference)	1.00 (reference)	1.00 (reference)
	50th	512–728 kcal	2.20 (1.08, 4.51) *	2.38 (1.11, 5.13) *	1.86 (0.65, 5.31)	2.79 (0.81, 9.69)
	75th	729–892 kcal	2.04 (0.99, 4.19)	2.33 (1.03, 5.32) *	3.69 (1.18, 11.58) *	1.59 (0.43, 5.90)
	100th	>892 kcal	3.75 (1.82, 7.77) ***	4.45 (1.71, 11.56) **	4.85 (1.44, 16.39) *	4.53 (0.87, 23.58)
CHO Late EW	25th	<62.1 g	1.00 (reference)	1.00 (reference)	1.00 (reference)	1.00 (reference)
	50th	62.1–85.0 g	1.99 (0.98, 4.06)	2.05 (0.96, 4.39)	2.00 (0.69, 5.80)	1.85 (0.58, 5.85)
	75th	85.1–109.5 g	1.85 (0.90, 3.77)	1.98 (0.89, 4.37)	2.35 (0.82, 6.74)	1.47 (0.42, 5.15)
	100th	>109.5 g	3.49 (1.69, 7.22) **	3.28 (1.31, 8.19) *	5.56 (1.47, 21.02) *	1.95 (0.50, 7.60)
Fat Late EW	25th	<21.3 g	1.00 (reference)	1.00 (reference)	1.00 (reference)	1.00 (reference)
	50th	21.3–28.0 g	1.32 (0.66, 2.65)	1.19 (0.56, 2.53)	0.98 (0.33, 2.93)	1.31 (0.43, 4.02)
	75th	28.1–36.4 g	1.79 (0.89, 3.57)	1.63 (0.74, 3.59)	2.29 (0.76, 6.84)	1.06 (0.32, 3.57)
	100th	>36.4 g	2.28 (1.14, 4.56) *	2.31 (0.99, 5.40)	2.09 (0.65, 6.68)	2.46 (0.67, 9.00)

* Significant *p* < 0.05, ** *p* < 0.01, *** *p* < 0.001 using logistic regression model. Dependent variable: MUO (1) MHO (0). Independent variable: EI early EW, CHO early EW, Protein early EW, EI late EW, CHO late EW, and Fat late EW. 1.0 (reference category). Model 1 adjusted for age and gender. Model 2 adjusted for age, gender, BMI, and total energy intake. Abbreviations: MUO, metabolically unhealthy obesity; OR, odds ratio; CI, confidence interval; EI, energy intake. CHO, carbohydrate; EW, eating window; M-type, morning chronotypes; E-type, evening chronotypes.

## Data Availability

The data presented in this study is a part of ongoing doctoral research of F.H.M. Hence, we could not publicly release the data. However, it is available upon request from the corresponding author (Z.A.M).

## Supplementary Materials

The following are available online at https://www.mdpi.com/article/10.3390/nu13114121/s1, Table S1: Lifestyle characteristics and dietary intake of MHO and MUO within each gender.
