# Supplementary material for: Do Temporal Eating Patterns Differ in Healthy versus Unhealthy Overweight/Obese Individuals?
Source: Nutrients. 2021 Nov 17;13(11):4121. doi: 10.3390/nu13114121 (PMC8618797; doi:10.3390/nu13114121)
Supplement: Supplementary file 1 [file nutrients-13-04121-s001.zip › nutrients-1385898-supplementary.pdf]

**Supplementary: Table S1** Lifestyle characteristics and dietary intake of MHO and MUO within each gender

| Characteristics | Men (n=173) MHO (n=37) Mean ± SE | Men (n=173) MUO (n=42) Mean ± SE | P value | Women (n=126) MHO (n=136) Mean ± SE | Women (n=126) MUO (n=84) Mean ± SE | P value |
|---|---|---|---|---|---|---|
| **Sleep traits** | | | | | | |
| Sleep duration workdays (hour) | 6.1 ± 0.2 | 6.1 ± 0.2 | 0.970 | 6.2 ± 0.1 | 6.3 ± 0.1 | 0.473 |
| Sleep duration free days (hour) | 6.2 ± 0.2 | 6.3 ± 0.2 | 0.816 | 6.9 ± 0.1 | 6.7 ± 0.1 | 0.306 |
| MSW (local time) | 02:38 ± 0:42 | 02:38 ± 0:39 | 0.277 | 02:25 ± 0:47 | 02:31 ± 0:41 | 0.398 |
| MSF (local time) | 03:08 ± 0:58 | 03:02 ± 0:59 | 0.453 | 02:49 ± 1:01 | 02:55 ± 0:54 | 0.722 |
| Social jetlag (minute) | 28.3 ± 6.1 | 28.4 ± 5.7 | 0.991 | 34.8 ± 3.0 | 26.9 ± 3.8 | 0.110 |
| **Physical activity** | | | | | | |
| MET | 2869.7 ± 513.9 | 1747.0 ± 480.5 | 0.125 | 1754.9 ± 282.1 | 2851.0 ± 359.7 | 0.020* |
| **Night eating syndrome** | | | | | | |
| Score | 9.6 ± 0.8 | 10.3 ± 0.7 | 0.565 | 10.4 ± 0.4 | 10.7 ± 0.6 | 0.669 |
| **Total dietary intakes** | | | | | | |
| Energy intake (kcal/day) | 2063 ± 89 | 2116 ± 83 | 0.670 | 1730 ± 35 | 1818 ± 45 | 0.128 |
| Energy intake (kcal/kg BW) | 23.4 ± 1.1 | 23.3 ± 1.0 | 0.917 | 22.9 ± 0.5 | 23.7 ± 0.6 | 0.293 |
| CHO (g/day) | 256.6 ± 12.9 | 267.7 ± 12.1 | 0.546 | 209.2 ± 4.8 | 219.8 ± 6.2 | 0.189 |
| % E from CHO | 49.6 ± 1.0 | 49.9 ± 0.9 | 0.848 | 48.6 ± 0.6 | 47.8 ± 0.8 | 0.441 |
| Protein (g/day) | 76.5 ± 2.9 | 74.7 ± 2.8 | 0.664 | 66.7 ± 1.4 | 69.4 ± 1.8 | 0.230 |
| % E from protein | 15.0 ± 0.5 | 14.6 ± 0.5 | 0.531 | 15.6 ± 0.2 | 15.4 ± 0.3 | 0.635 |
| Fat (g/day) | 81.2 ± 3.8 | 82.9 ± 3.5 | 0.739 | 69.6 ± 1.8 | 73.5 ± 2.3 | 0.189 |
| % E from fat | 35.4 ± 0.7 | 35.5 ± 0.7 | 0.901 | 35.8 ± 0.5 | 36.2 ± 0.6 | 0.654 |
| **Meal timing** | | | | | | |
| First mealtime (local time) | 08:33 ± 1:14 | 08:05 ± 0:50 | 0.292 | 08:31 ± 0:45 | 08:27 ± 0:46 | 0.375 |
| Last mealtime (local time) | 21:02 ± 1:37 | 20:17 ± 1:25 | 0.183 | 20:35 ± 1:29 | 20:27 ± 1:29 | 0.357 |
| Midpoint of eating (local time) | 14:48 ± 1:11 | 14:12 ± 0:52 | 0.135 | 14:33 ± 0:51 | 14:25 ± 0:54 | 0.205 |
| Elapse time between last meal and sleep onset (hour) | 2.8 ± 0.3 | 3.4 ± 0.3 | 0.160 | 2.8 ± 0.1 | 3.1 ± 0.2 | 0.262 |
| Total eating window (hour) | 12.5 ± 0.3 | 12.2 ± 0.3 | 0.552 | 12.1 ± 0.1 | 12.0 ± 0.2 | 0.745 |

*Significant p<0.05 using ANCOVA test adjusted to age, and BMI. Abbreviations: MHO, metabolically healthy obesity; MUO, metabolically unhealthy obesity; SE, standard error; BW, body weight; CHO, carbohydrate; % E, percentage energy; MSW, midpoint of sleep on work days; MSF, midpoint of sleep on free days; MET, metabolic equivalent
